# H3N2v and Other Influenza Epidemic Risk Based on Age-Specific Estimates of Sero-Protection and Contact Network Interactions

**DOI:** 10.1371/journal.pone.0054015

**Published:** 2013-01-11

**Authors:** Danuta M. Skowronski, Flavia S. Moser, Naveed Z. Janjua, Bahman Davoudi, Krista M. English, Dale Purych, Martin Petric, Babak Pourbohloul

**Affiliations:** 1 Communicable Disease Prevention and Control Services, British Columbia Centre for Disease Control, Vancouver, Canada; 2 School of Population and Public Health, University of British Columbia, Vancouver, Canada; 3 BC Biomedical Laboratories Ltd, Surrey, Canada; 4 Public Health Microbiology and Reference Laboratory, British Columbia Centre for Disease Control, Vancouver, Canada; 5 Department of Pathology and Laboratory Medicine, University of British Columbia, Vancouver, Canada; Melbourne School of Population Health, Australia

## Abstract

Cases of a novel swine-origin influenza A(H3N2) variant (H3N2v) have recently been identified in the US, primarily among children. We estimated potential epidemic attack rates (ARs) based on age-specific estimates of sero-susceptibility and social interactions. A contact network model previously established for the Greater Vancouver Area (GVA), Canada was used to estimate average epidemic (infection) ARs for the emerging H3N2v and comparator viruses (H1N1pdm09 and an extinguished H3N2 seasonal strain) based on typical influenza characteristics, basic reproduction number (R_0_), and effective contacts taking into account age-specific sero-protection rates (SPRs). SPRs were assessed in sera collected from the GVA in 2009 or earlier (pre-H1N1pdm09) and fall 2010 (post-H1N1pdm09, seasonal A/Brisbane/10/2007(H3N2), and H3N2v) by hemagglutination inhibition (HI) assay. SPR was assigned per convention based on proportion with HI antibody titre ≥40 (SPR40). Recognizing that the HI titre ≥40 was established as the 50%sero-protective threshold we also explored for ½SPR40, SPR80 and a blended gradient defined as: ¼SPR20, ½SPR40, ¾SPR80, SPR160. Base case analysis assumed R_0_ = 1.40, but we also explored R_0_ as high as 1.80. With R_0_ = 1.40 and SPR40, simulated ARs were well aligned with field observations for H1N1pdm09 incidence (AR: 32%), sporadic detections without a third epidemic wave post-H1N1pdm09 (negligible AR<0.1%) as well as A/Brisbane/10/2007(H3N2) seasonal strain extinction and antigenic drift replacement (negligible AR<0.1%). Simulated AR for the novel swine-origin H3N2v was 6%, highest in children 6–11years (16%). However, with modification to SPR thresholds per above, H3N2v AR ≥20% became possible. At SPR40, H3N2v AR ≥10%, ≥15% or ≥30%, occur if R_0_≥1.48, ≥1.56 or ≥1.86, respectively. Based on conventional assumptions, the novel swine-origin H3N2v does not currently pose a substantial pandemic threat. If H3N2v epidemics do occur, overall community ARs are unlikely to exceed typical seasonal influenza experience. However risk assessment may change with time and depends crucially upon the validation of epidemiological features of influenza, notably the serologic correlate of protection and R_0_.

## Introduction

Influenza virus reassortment events in swine have been implicated in the origin of previous pandemics of the 20^th^ century and the first pandemic of the 21^st^ century (2009) [1]–[7]. The 2009 pandemic H1N1 virus (H1N1pdm09) was a complex retro-reassortment inasmuch as its surface hemagglutinin (HA) protein, to which antibody protection is primarily directed, bears closest resemblance to the historic human H1 of 1918 and is antigenically more distant from modern H1 strains [6]–[8]. Consistent with this ancestral phylogeny, sero-surveys have shown that pre-pandemic susceptibility to H1N1pdm09 was virtually universal across all age groups except the very old [8]–[15]. Very old individuals who had been exposed to 1918-like H1 strains in early childhood may have benefitted decades later from that robust priming experience through cross-protective antibody against H1N1pdm09 [8]. Subsequent to the fall 2009 pandemic wave, H1N1pdm09 protection across the population was dramatically altered with substantial levels of infection- and/or vaccine-induced antibody also found in young children and adults [13]–[18]. In the Greater Vancouver Area (GVA) of British Columbia, Canada, low-level detections but no large-scale epidemics due to H1N1pdm09 were observed during the subsequent 2010–11 and 2011–12 seasons with return to a mix of circulating strains, predominantly seasonal H3N2 and B [19] as also observed elsewhere in Canada [20], [21] and the United States (US) [22], [23].

During the latter half of 2011 and the first half of 2012, thirteen human infections due to another newly emerging swine-origin influenza virus of the H3N2 subtype were identified across six US states, primarily among children [24]–[28]. This H3N2 variant has been designated H3N2v by the World Health Organization (WHO) [29]. Of these 13 initial H3N2v detections, three were hospitalized and six had no swine exposure, raising concern about unrecognized human-to-human transmission, a potential recently confirmed in ferret studies [30]. Between July and end of September 2012, 306 additional H3N2v detections were reported in outbreaks across ten US states, including 16 hospitalizations and one death. Most of these cases were linked to swine exposure through agricultural fairs with only limited human-to-human transmission identified [31]. With fall-winter return to school, however, concern about further possible propagation was raised [31].

Phylogenetic analysis indicates that the HA of H3N2v descended from a human H3N2 ancestor virus, with the matrix protein acquired from H1N1pdm09 [32], [33]. This ancestral strain of H3N2v circulated during a period less distant in time from that of H1N1pdm09, bearing closest resemblance to human H3N2 viruses from the mid-1990s [30], [32], [33]. Consistent with this more recent ancestral phylogeny, sero-surveys conducted in Canada [33], [34], the US [35] and Norway [36] show that the human population is not entirely immunologically naïve to H3N2v, with age-related variation in sero-protection, highest in teens and young adults who were likely primed with related H3N2 strains in childhood [34]. Conversely, younger children and older adults show broader susceptibility.

This complex profile of population immunity by age for H3N2v makes it difficult to predict the likelihood of community-wide epidemic spread. Although the population is not entirely susceptible, disproportionate vulnerability in children with extensive social contacts could amplify virus spread sufficiently to overcome the partial barrier of protection in other age groups [37]. To better inform risk assessment we have thus incorporated available sero-survey findings into an established contact network model that can simultaneously account for individual-level variation in susceptibility and social interactions by age. We use this contact network to assess the potential for large-scale epidemic spread of H3N2v. For added context and interpretation we compare against model-generated predictions and actual field observations for the successfully-propagated H1N1pdm09 virus and an extinguished H3N2 human seasonal strain, as well as community attack rates for seasonal influenza typically cited in the range of 5–15% during epidemic seasons [38].

## Methods

Initial infections may result in limited human-to-human transmission before becoming extinguished; we refer to such events as *small-scale outbreaks*. Conversely, we refer to sustained transmission within a community as an *epidemic* or *large-scale outbreak*.

Our main objective was to assess epidemic risk in the event sustained H3N2v spread occurs and no interventions are applied. Viewing epidemics as events conducted through networks of interpersonal contacts, the outcome endpoint can be quantified as the fraction of all individuals in the network/population to which infection is transmitted–also known as the epidemic (infection) attack rate (AR). Once the epidemic becomes established within a community, network theory ensures that the AR remains a robust indicator of spread for a specific disease, irrespective of the number of initial cases or the subsequent configuration of transmission pathways [39]. We thus summarize the main outcome endpoint for each of the virus/immunity scenarios by the epidemic (infection) AR.

### Model Structure

An established contact network model previously structured for the GVA was used. Details, including sensitivity analyses around the main parameters, have been described previously [39]–[44], and are summarized here.

Individuals within the population are represented in the model as a network of nodes and their relevant contact interactions as links or edges. Nodes are assigned an age and household membership based on census statistics. Edges are defined using age-related interactions relevant to influenza transmission during a typical week and are modeled using distributions specific to the same geographical region identified from multiple sources including household size, employment, school attendance, commuter patterns, care facility residence, shopping mall visits, etc. [40], [42]. The resulting network includes 2.2 million nodes, and approximately 18 million edges appropriate to the GVA.

### Transmissibility Effects

The likelihood of small-scale versus large-scale outbreaks depends upon pathogen-specific *transmissibility* (denoted ***T***). In the context of contact network models, ***T*** is defined as the probability that a person, while infectious, transmits the infection to a susceptible contact. Transmissibility is closely related to the familiar epidemiologic concept, the *basic reproduction number*, ***R_0_*** [39], [45]. ***R_0_*** is the expected number of secondary cases per primary infected case in a *totally* susceptible population. If ***R_0_*** is <1, only small-scale outbreaks will occur; if ***R_0_*** is >1, epidemic spread becomes possible. Transition between these two scenarios occurs at ***R_0_*** = 1.

### Susceptibility Effects

In a network model, pathogens can only relay from an infectious individual to susceptible contacts. The model allows a fraction of individuals (i.e. nodes) by age to be immune. Of note, when the immunity profile changes network contributions, it also acts upon ***R_0_*** because the population is no longer completely susceptible. Techniques inherent in the contact network approach allow the modifying effects of immunity on the number of secondary cases generated reflecting an *effective reproduction number,* ***R_eff_***.

### Immunity Scenarios

Immunity profiles by age were based on sero-survey estimates derived for the GVA according to protocols previously reported and approved by the University of British Columbia Research Ethics Board [8], [16], [34]. Briefly, for each virus, the proportion sero-protected by age was based on ∼1000 anonymized community-based residual sera assembled as a convenience sample of ∼100 sera per decade of life (ranging <1–100 years old) collected from the GVA, presented here according to model-relevant age categories. Sera were tested for strain-specific antibody by the hemagglutination inhibition (HI) assay [8], [16], [34].

Sera tested for pre-existing antibody to H3N2v (A/Indiana/10/2011-like) and post-circulation antibody to an extinguished human seasonal H3N2 strain had been collected in fall 2010. The extinguished seasonal H3N2 strain selected was A/Brisbane/10/2007-like (hereafter “post-Brisbane”) because it was the last dominant H3N2 strain to circulate prior to fall 2010 when immune pressure led to its evolution to a subsequent antigenically-distinct descendant strain [46].

Pre-pandemic sero-protection for A(H1N1)pdm09 virus (A/California/07/2009-like) was assessed in sera collected in 2009 and earlier (“pre-H1N1pdm09”) [8]. To assess the impact of post-pandemic sero-protection against H1N1pdm09 (“post-H1N1pdm09”), the sera collected in fall 2010 were also tested for antibody to that strain; these sera would mostly reflect H1N1pdm09 sero-protection acquired through infection or immunization during fall 2009.

Sero-protection was defined as the proportion meeting or exceeding the classically-accepted threshold HI antibody titre ≥40 [47]–[50]. Although this is widely interpreted as the 100% sero-protection threshold, the HI titre ≥40 has in fact been established at the 50% sero-protective level–i.e. the titre at which half of individuals are protected from infection under various experimental or analytical conditions [49]. We therefore explored endpoints assuming that titre ≥40 defines the 100%sero-protection rate (SPR) and additionally assuming titre ≥40 represents the 50%sero-protection rate (**½**SPR40) or that the 100% sero-protection rate is defined instead at titre ≥80 (SPR80). We also explored based on a blended composite of sero-protection assuming a categorical gradient of immunity by titre assigned as **½**SPR40 (i.e. 50% of those with titre 40–79 protected), ¾SPR80, (i.e. 75% of those with titre 80–159 protected) and SPR160 (i.e. all with titre 160 or higher protected) with and without also incorporating ¼SPR20 (i.e. 25% with titre 20–39 protected).

Virus/immunity scenarios explored are defined in Table 1. The main outcome of interest was for H3N2v age-specific immunity defined by SPR40. Other virus/immunity scenarios were explored for comparison and interpreted in the context of observed surveillance patterns.

**Table 1:** Main input parameter values for Greater Vancouver Area (GVA) contact network model.

Variable		Value (range)	Source
Population size by age	Age Group (years)		[2006 Statistics Canada^a^]
	<2	47,755	
	2–5	85,396	
	6–11	141,215	
	12–17	162,159	
	18–24	222,032	
	25–44	686,771	
	45–64	580,141	
	≥65	271,455	
	**Total**	**2,196,852**	
GVA model parameters			[39]–[40], [42]–[44],2006 Statistics Canada^a^
**Shared virus parameters** b			
Latent period		2 days (1–3 days)	[52]
Infectious period		Randomly assigned1–5 days (1–7 days)	[51], [52]
Basic Reproduction Number (***R_0_***)		1.40, 1.80	[52]–[55]
Number of initial infections		10	
**Virus/Immunity Scenario**	**Example**		**Label**
1. No immunity	A novel influenza virus emerging into a totally susceptible population		“No Immunity”
2. Virtually no immunity	Pre-H1N1pdm09 age-specific immunity		“Pre-H1N1pdm09 (SPR40)”^c^
3. High proportion immune	Post-H1N1pdm09 age-specific immunity		“Post-H1N1pdm09 (SPR40)”^c^
4. Proportion immune	H3N2v age-specific immunity		“H3N2v (SPR40)”^c^
5. Proportion immune	H3N2v age-specific immunity		“H3N2v (**½**SPR40)”^d^
6. Proportion immune	H3N2v age-specific immunity		“H3N2v (SPR80)”^e^
7. High proportion immune	Post-epidemic seasonal H3N2 (Brisbane/10/2007) age-specific immunity		“Post-Brisbane (SPR40)”^c^

HI = hemagglutination inhibition assay; SPR = Sero-protection rate – defined as the proportion (%) considered sero-protected on the basis of having met or exceeded the specified antibody titre threshold.

**pre-H1N1pdm09**: 2009 H1N1 pandemic virus; SPR based on PRE-pandemic antibody levels in 2009 or earlier.

**post-H1N1pdm09**: 2009 H1N1 pandemic virus; SPR based on POST-pandemic antibody levels fall 2010.

**H3N2v:** swine-origin H3N2 variant strain; SPR based on antibody levels in fall 2010.

**post-Brisbane**: a seasonal human influenza H3N2 virus; SPR based on antibody levels fall 2010.

a Statistics Canada 2006 Community Profiles [http://www12.statcan.ca/census-recensement/2006/dp-pd/prof/92-591/index.cfm?Lang=E].

b The same typical seasonal influenza parameter values were applied to each of the three influenza viruses assessed, recognizing their human influenza virus ancestral origins.

c Based on 100% SPR defined at HI titre ≥40; ^d^Based on 50%SPR defined at HI titre ≥40; ^e^100%SPR defined at HI titre ≥80.

Note that a blended composite of sero-protection based on a gradient of immunity defined as **¼**SPR20, ½SPR40, ¾SPR80 and 100% SPR160 was also explored (see Table S1 and narrative).

### Model Simulations

The original GVA contact network was modified to account for age-based immunity profiles. This was achieved by randomly removing a fraction of nodes in each age bracket to comply with virus-specific immunity distributions, with nodes assigned dichotomously as immune or susceptible. In this way, individuals maintain the same underlying pattern of social linkage, but a proportion of network edges are rendered non-contributory by nodal immunity and are thus removed from network play. The effect of removing individuals based on nodal immunity and non-contributory edges can be represented through average degree, i.e. the number of potential infection-causing interpersonal contacts that individuals may have during the week. These combined immunity/network effects are illustrated in Figures S1 and S2.

To account for stochastic (i.e. probabilistic or random) effects, we created an ensemble of contact networks for each scenario. We used these network ensembles to perform an array of computer simulations wherein individuals are classified according to classic SEIR disease progression as *Susceptible, Exposed, Infectious, Recovered/Removed* [51]. Individuals remain in the *Susceptible* state until they acquire infection through one of their links. At this point, they enter the *Exposed* state, where they are assumed to be infected but cannot infect others and this interval is called the latent period. They then transition to the *Infectious* state, where they potentially infect susceptible individuals linked to them. After the infectious period, individuals enter the *Recovered/Removed* state, where they no longer contribute to disease transmission. Those with *pre-existing* vaccine- or infection-induced immunity are also assigned at the outset to the *Recovered/Removed* state.

Recognizing that transmission remains probabilistic in nature we ran multiple simulations. To arrive at final point estimates we averaged as the mean across all simulations for each scenario.

### Model Assumptions

Main parameter input values and ranges included in sensitivity analyses are outlined in Table 1. We assumed the same parameter values for each virus/immunity scenario, applying typical seasonal influenza estimates as previously described [44], [52]. We assumed a latent period of 2 days (ranged 1–3 days) [45], an infectious period randomly varying between 1–5 days (extended to 1–7 days in sensitivity analysis) [53] and 10 initial infections. Measured estimates of virus-specific sero-protection by age for the various scenarios are presented in Table 2 (SPR40, ½SPR40, SPR80) and in Table S1 (blended composite of sero-protection) [8], [16], [34]. Our main analysis refers to SPR40 and an ***R_0_*** = 1.40 but we also explored for ***R_0_*** = 1.80 [52]–[55].

**Table 2:** Proportion (%) considered immune by age category, virus and antibody threshold.

	Scenario (SPR by virus and threshold titre with (95% Confidence Intervals))						
Age	No	pre-	post-				post-
Categories	Immunity	H1N1pdm09	H1N1pdm09	H3N2v	H3N2v	H3N2v	Brisbane
(Years)		(SPR40) [8]	(SPR40)^1^	(SPR40) [34]	(½SPR40) [34]	(SPR80)^2^ [34]	(SPR40) [34]
**<2**	0	0.0	46.5 (34.9–58.1)	0.0	0.0	0.0	8.9 (0.6–17.2)
**2–5**	0	1.1 (0.0–3.3)	71.0 (63.0–79.0)	0.0	0.0	0.0	43.8 (34.3–53.3)
**6–11**	0	1.1 (0.0–3.1)	59.6 (49.3–69.8)	17.2 (9.3–25.2)	8.6 (4.6–12.6)	3.4 (0.0–7.3)	78.2 (69.5–86.9)
**12–17**	0	10.1 (3.0–17.3)	60.4 (47.2–73.6)	42.3 (28.9–55.8)	21.2 (14.4–27.9)	9.6 (1.6–17.6)	57.7 (44.2–71.1)
**18–24**	0	1.6 (0.0–4.8)	28.2 (18.2–38.2)	60.3 (49.4–71.1)	30.2 (24.7–35.6)	23.1 (13.7–32.4)	38.5 (27.6–49.3)
**25–44**	0	8.8 (4.9–12.7)	30.8 (24.5–37.2)	35.7 (29.0–42.3)	17.8 (14.5–21.2)	10.1 (5.9–14.2)	29.1 (22.8–35.5)
**45–64**	0	4.6 (1.7–7.6)	14.9 (10.0–19.9)	6.5 (3.1–9.9)	3.2 (1.5–4.9)	0.0	22.4 (16.6–28.2)
**65+**	0	43.1 (37–49.2)	38.1^3^ (33.1–43.2)	19.8 (15.6–24.0)	9.9 (7.8–12.0)	4.0 (1.9–6.1)	43.6 (38.3–48.8)
**Overall** 4	0	10.3 (8.6–12.1)	33.2 (30.2–36.2)	25.3 (22.6–28.0)	12.6 (11.3–14.0)	6.7 (5.0–8.5)	34.5 (31.4–37.5)

**SPR** = Sero-protection rate – defined as the proportion (%) considered sero-protected on the basis of having met or exceeded the specified antibody titre threshold.

**pre-H1N1pdm09**: 2009 H1N1 pandemic virus; SPR presented based on PRE-pandemic antibody levels measured in 2009 or earlier.

**post-H1N1pdm09**: 2009 H1N1 pandemic virus; SPR presented based on POST-pandemic antibody levels measured in fall 2010.

**H3N2v**: swine-origin H3N2 variant strain; SPR presented based on antibody levels measured in sera collected in fall 2010.

**post-Brisbane:** a contemporary seasonal human influenza H3N2 virus; SPR presented based on post-circulation antibody levels in sera collected in fall 2010.

**SPR40**: the proportion considered sero-protected according to the standard hemagglutination inhibition (HI) titre threshold of 40.

**½SPR40**: assumes half the individuals meeting SPR40 are considered sero-protected.

**SPR80**: the proportion considered sero-protected according to a hemagglutination inhibition (HI) titre threshold of 80.

Note that a blended composite of sero-protection based on a gradient of immunity defined as **¼**SPR20, ½SPR40, ¾SPR80 and 100% SPR160 was also explored (see Table S1 and narrative).

1 Author unpublished data.

2 The proportion with titre ≥160 was 2% (95%CI 1–3%) overall, highest in those 18–24 years of age (10%; 95%CI 4–17%).

3 Lower post-pandemic estimate for this age group within expected error of laboratory assay method and sampling variability.

4 Overall age-standardized SPRs are displayed for interest but were not used in generating model-based AR estimates.

## Results

### Epidemic Likelihood

**Figure 1:**
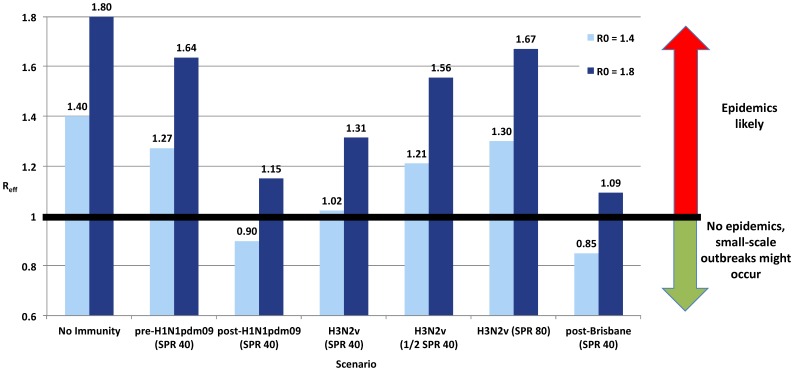
Effective reproduction number (*R_eff_*) according to various scenarios of population immunity by initial basic reproduction number (*R_0_*) assumed. **SPR** = Sero-protection rate – defined as the proportion (%) considered sero-protected on the basis of having met or exceeded the specified antibody titre threshold; **pre-H1N1pdm09:** 2009 H1N1 pandemic virus; SPR presented based on PRE-pandemic antibody levels measured in 2009 or earlier; **post-H1N1pdm09:** 2009 H1N1 pandemic virus; SPR presented based on POST-pandemic antibody levels measured in fall 2010; **H3N2v:** swine-origin H3N2 variant strain; SPR presented based on antibody levels measured in sera collected in fall 2010; **post-Brisbane:** a contemporary seasonal human influenza H3N2 virus; SPR presented based post-circulation antibody levels in sera collected in fall 2010; **SPR40:** the proportion considered sero-protected according to the standard hemagglutination inhibition (HI) titre threshold of 40; **½SPR40:** assumes half the individuals meeting SPR40 are considered sero-protected; **SPR80:** the proportion considered sero-protected according to a hemagglutination inhibition (HI) titre threshold of 80.

Figure 1 illustrates the impact of altering underlying assumptions of population immunity on ***R_0_*** expressed through ***R_eff_***. Assuming ***R_0_*** = 1.40 and applying the measured age-related immunity profile for H3N2v at SPR40, the ***R_eff_*** approaches one (1.02) and the likelihood of epidemic spread is greatly diminished. However, this effect is sensitive to the sero-protective threshold used to define immunity. At **½**SPR40 or SPR80, ***R_eff_*** for H3N2v (1.21 and 1.30, respectively) approaches or exceeds that of pre-H1N1pdm09 at SPR40 (1.27). An ***R_eff_***>1 for the pre-H1N1pdm09 age-specific immunity profile is consistent with its successful pandemic spread. The ***R_eff_***<1 for post-H1N1pdm09 (0.90) is consistent with absence of large-scale epidemic activity in the GVA in subsequent seasons [19]. Also consistent with laboratory surveillance and seasonal H3N2 evolution [19], [46], ***R_eff_*** for the post-Brisbane immunity profile is <1 (0.85).

Conversely, applying an assumption of higher ***R_0_*** = 1.80 suggests epidemic potential across virus/immunity scenarios (Figure 1), including a post-H1N1pdm09 third wave which was not observed in any major urban area of Canada [19]–[21] or the US [22], [23]. We emphasize findings at ***R_0_*** = 1.40.

### Epidemic Attack Rates

**Figure 2:**
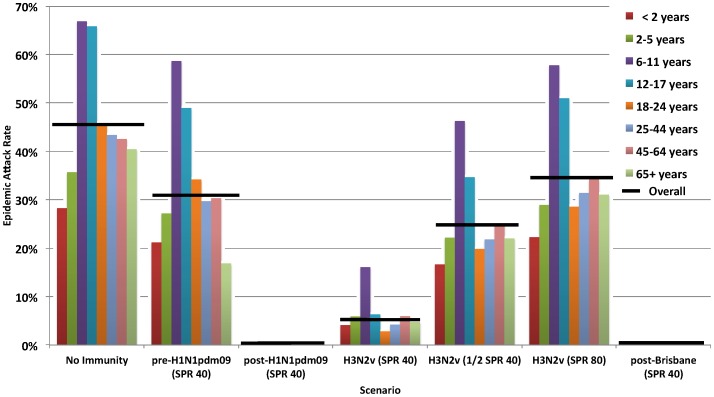
Age-stratified epidemic attack rates by scenario and assumed basic reproduction number (*R_0_* = 1.40). SPR = Sero-protection rate – defined as the proportion (%) considered sero-protected on the basis of having met or exceeded the specified antibody titre threshold; **pre-H1N1pdm09:** 2009 H1N1 pandemic virus; SPR presented based on PRE-pandemic antibody levels measured in 2009 or earlier; **post-H1N1pdm09:** 2009 H1N1 pandemic virus; SPR presented based on POST-pandemic antibody levels measured in fall 2010; **H3N2v:** swine-origin H3N2 variant strain; SPR presented based on antibody levels measured in sera collected in fall 2010; **post-Brisbane:** a contemporary seasonal human influenza H3N2 virus; SPR presented based post-circulation antibody levels in sera collected in fall 2010; **SPR40:** the proportion considered sero-protected according to the standard hemagglutination inhibition (HI) titre threshold of 40; **½SPR40:** assumes half the individuals meeting SPR40 are considered sero-protected; **SPR80:** the proportion considered sero-protected according to a hemagglutination inhibition (HI) titre threshold of 80 Overall attack rates are indicated by the horizontal line. Based on simulations using age-specific parameters, these overall attack rates were derived as the total number of infections divided by the total population size.

Figure 2 shows estimates of overall and age-specific ARs for ***R_0_*** = 1.40. Assuming no immunity in the population, our model indicates that a novel influenza virus with the characteristics we have defined may achieve an overall epidemic AR of 45%.

With ≤10% of the pediatric and adult population immune but about 40% of the elderly protected, (as per the pre-H1N1pdm09 scenario based on SPR40), the model-generated AR is 32%(Figure 2), comparable if slightly higher than measured AR estimates from Canada ranging 20–30% [15]. At pre-H1N1pdm09 immunity defined instead at **½**SPR40 and SPR80, ARs are higher at 38% and 41%, respectively. Using a blended composite of sero-protection assigned as **½**SPR40, ¾SPR80, and SPR160, with or without ¼SPR20, the model-generated pandemic H1N1 AR is also higher at 37%. However, these differences in AR from SPR40 include assumptions of reduced immunity and higher AR in the elderly–a pattern that was not observed in reality. We emphasize findings at SPR40.

For the post-H1N1pdm09 and the post-Brisbane scenarios based on ***R_0_*** = 1.40 and SPR40, average epidemic ARs are negligible (<0.1%), consistent with surveillance findings, but again substantially higher for both at **½**SPR40 (18% and 14%, respectively), SPR80 (23% and 21%, respectively) or based on the blended composite of sero-protection (7–8% and 3–4%, respectively).

Increasing ***R_0_*** = 1.80 also leads to very different and implausible ARs at SPR40, **½**SPR40 and SPR80 for pre-H1N1pdm09 (53%, 60% and 63%, respectively), post-H1N1pdm09 (15%, 43% and 48%, respectively) and post-Brisbane (9%, 40% and 46%, respectively) scenarios. None of these estimates based on ***R_0_*** = 1.80 is consistent with field experience.

For the novel H3N2v scenario, simulations based on ***R_0_*** = 1.40 and SPR40 predict AR of 6%. Applying the upper and lower limits of the 95% confidence intervals for the age-specific H3N2v SPR40 shown in Table 2, the overall ARs range from negligible (<0.1%) to 15%. These estimates are higher at **½**SPR40 (25%), SPR80 (35%), or based on the blended composite (20–21%).

**Figure 3:**
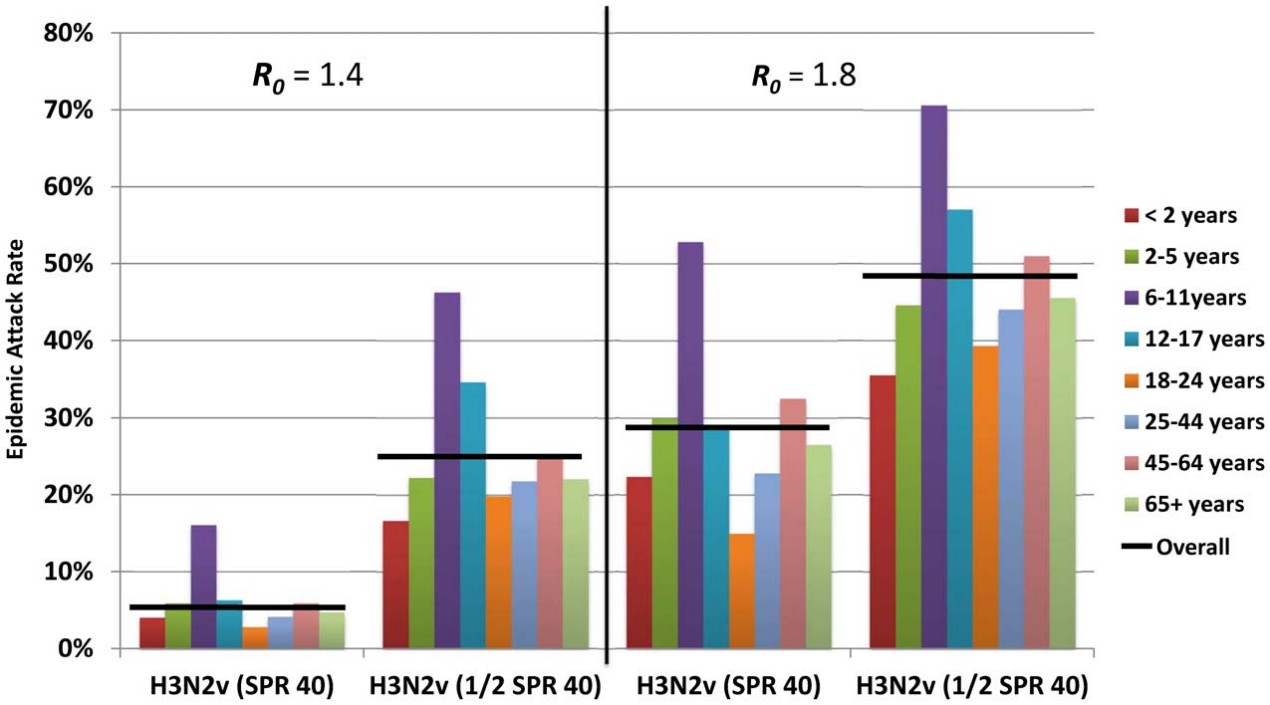
Age-stratified epidemic attack rates by scenario and assumed basic reproduction number (*R_0_*). SPR = Sero-protection rate – defined as the proportion (%) considered sero-protected on the basis of having met or exceeded the specified antibody titre threshold; **H3N2v:** swine-origin H3N2 variant strain; SPR presented based on antibody levels measured in sera collected in fall 2010; **SPR40:** the proportion considered sero-protected according to the standard hemagglutination inhibition (HI) titre threshold of 40; **½SPR40:** assumes half the individuals meeting SPR40 are considered sero-protected Overall attack rates are indicated by the horizontal line. Based on simulations using age-specific parameters, these overall attack rates were derived as the total number of infections divided by the total population size.

To reach overall ARs ≥10%, ≥15% or ≥30% for H3N2v based on SPR40, the ***R_0_*** would have to be ≥1.48, ≥1.56 or ≥1.86, respectively. At the upper range, if ***R_0_*** = 1.80 then H3N2v AR are 28%(SPR40), 48%(**½**SPR40) and 61%(SPR80)(Figure 3).

Across all immunity scenarios, ARs are highest among young school-age children 6–11 years old(Figure2). In that age group at ***R_0_*** = 1.40 and SPR40, an H3N2v AR of 16% may occur while in children 2–5 years or 12–17 years, lower ARs of 6% reflect the combined influences of differential susceptibility and social interactions. At **½**SPR40 or SPR80, corresponding ARs are 46% and 58% in children 6–11 years of age and at ***R_0_*** = 1.80, ARs range 50–80% in children 6–11 years of age. At ***R_0_*** = 1.48 and SPR40 (overall AR 10%), the AR for H3N2v in children 6–11 years of age could reach 27% and in those 12–17 years old would be 12%.

## Discussion

Recent zoonotic infections due to a swine-origin H3N2 influenza variant have prompted concern regarding its epidemic potential [24]–[31]. Phylogenetic analysis indicates H3N2v shares a common human influenza ancestor from the mid-1990s [32], [33]. As such, inherent transmissibility comparable to other human reassortant strains is a reasonable assumption, recently confirmed in ferret studies [30]. Population susceptibility and contact opportunities therefore become critical determinants of spread. Immunity profiles indicate a proportion of teens and young adults may already be protected [33]–[36]. However, extensive social contacts and flanking susceptibility in younger children and older adults make it less straightforward to predict the probability of an epidemic. Here we have simulated that risk assessment based on measured sero-protection by age applied to an established contact network model. By simultaneously incorporating susceptibility and social interactions we are able to quantify the epidemic risk, and also frame its interpretation in relation to recent pandemic as well as typical seasonal influenza experience.

Our approach has several strengths. Unlike other models predicated on assumptions of homogeneous mixing and susceptibility patterns, we have been able to simultaneously vary interactions and immunity at the individual level. Age-related immunity assumptions were based on measured sero-protection and interactions were based on census data for the same GVA locale. Sero-protection measured in the same way for the same general area across several influenza viruses of interest supported model simulations that could then also be compared against field observations for several pandemic and seasonal viruses. This provides context for interpreting our H3N2v-specific predictions.

Based on ***R_0_*** = 1.40 and SPR40, model-simulated predictions of pandemic H1N1 AR (32%) were comparable if slightly higher than the global estimate of pandemic H1N1 incidence based on meta-analysis of serologic studies conducted across multiple countries and continents (24%; 95%CI 20–27%) [56], comparable also to estimates for Canada ranging 20–30% [15]. Simulated ARs for post-H1N1pdm09 were also aligned with surveillance observations indicating sporadic detections but no third pandemic wave thereafter in the GVA or other major urban areas of Canada [19]–[21] or the US [22], [23]. Finally, model-estimated ARs were also compatible with extinction of the seasonal H3N2 Brisbane virus and its subsequent replacement by an antigenically-distinct seasonal H3N2 drift strain [46].

Applying ***R_0_*** = 1.40 and SPR40 for the novel H3N2v to the same network model, we estimated an overall AR of 6%, higher in young school-age children (16%). These findings represent the accumulated epidemic AR over a one-year period although seasonal variability in influenza transmission, not incorporated here, may influence how/when epidemic activity is clustered within that period. As a general principle of the undirected contact network we used the probability of an epidemic is equivalent to the overall epidemic AR [41]. Consistent with this, simulations based on ***R_0_*** = 1.40 and SPR40 revealed a current probability of epidemic spread of H3N2v of 6% in the context of the urban characteristics, social interactions and age-specific immunity profiles we assigned. In that context, the AR of 6% for H3N2v may be interpreted as intermediate between that of seasonal H3N2 strain extinction and widespread pandemic H1N1 propagation, greater also than the AR associated with low-level H1N1pdm09 detections post-2009 in the GVA and within, but at the lower end of, typical seasonal influenza experience, for which community AR in the range of 5–15% are commonly cited for epidemic seasons [38].

Although our model simulations suggest that the pandemic risk associated with H3N2v is not substantial, our simulations also indicate that young children would suffer higher infection rates in the event of sustained spread, a pattern also recognized with seasonal influenza [38]. A proportion of these pediatric infections may further experience severe complications such as hospitalization or death, not assessed here but relevant to further risk assessment and analysis. Similarly, elderly people may be at lower infection risk but higher individual risk of severe complications if infected, a pattern also recognized during the 2009 H1N1 pandemic [8]. H3N2 subtype seasons in general tend to be more severe than other subtypes and in particular, elderly people suffer disproportionately from H3N2 strains compared to other age groups [57]–[59]. Thus our findings should not be interpreted as reassurance against the need for specific vaccine; discussions regarding the development and strategic deployment of vaccine in the event of epidemic spread should continue.

There are additional insights to emphasize from our analysis, relevant to ongoing risk assessment. First, we highlight substantial differences in interpretation based on a two-fold adjustment to the defined serologic threshold for protection. Original studies to assess serologic correlates were based on the 50% sero-protective titre whereby half (rather than all) of a group was considered protected at that titre [49]. However, with common usage the 1:40 titre has been generally interpreted as the 100% sero-protective level. Sero-protection is generally dichotomized above or below 40 but a higher likelihood of protection at higher titre is recognized. A gradient of risk by antibody titre has not been empirically quantified [47]–[50]. By assigning no immunity below specified sero-protective thresholds, we over-estimate vulnerability and therefore risk. We have interpreted sero-survey results only in the context of dichotomized assignment of protection without considering other practical or theoretical implications, positive or negative, of low-level cross-reactive antibody. Because the HI assay does not necessarily represent functional antibody, other assays such as microneutralization, anti-neuraminidase or cytokine markers have been proposed but correlates or thresholds based on those techniques have not been established [47]–[50]. The dramatic difference in epidemic outcomes we report based on minor two-fold increase to the sero-protective threshold (SPR80), a single two-fold dilutional change within expected laboratory variation, highlights low-level HI antibody in the sera tested and underscores the potentially precarious nature of interpreting immunity. Few studies of serologic correlates have included children or older adults–higher thresholds have long been queried for the elderly and more recently also for young children [49], [60]. We did not vary sero-protective thresholds by age, but the low H3N2v titres and social interactions in young pre-school children and the elderly [8] suggest that selectively raising their thresholds for defining sero-protection is unlikely to have meaningfully altered conclusions. The clinical significance of cross-reactive titres or thresholds for protection against emerging zoonotic viruses is of further uncertainty. Variability with all antibody assays within and across laboratories is also generally understood. At a population level, recognizing other sources of variability, sero-protection estimates may be useful to gauge major trends, but where more detailed interpretation is important, greater precision is needed. On the population level, it is reassuring that results based on SPR40 were best aligned with field observations for comparator strains whereas those based on **½**SPR40, SPR80 or the blended composite of sero-protection were less consistent with surveillance patterns. We highlight the need for further epidemiologic or human challenge studies to define and validate serologic correlates of influenza protection by age relevant not only for H3N2v risk assessment, but also other influenza types/subtypes, emerging strains and public health applications.

Our findings also reveal substantial differences in interpretation across minor changes in ***R_0_***. Although a lower bound of ***R_0_*** for a novel reassortant virus cannot be defined *a priori*, previous estimates of the upper limit for pandemic influenza have spanned a very broad and high range (5–25) [45]. Conversely, more recent estimates of ***R_0_*** for influenza, specifically including H1N1pdm09, have converged within a lower and narrower range of 1.4–1.6 [42], [52]–[55], [61]. Our model shows the very different outcomes to be anticipated even across that narrower range. The assumption of ***R_0_*** = 1.40 modified through immunity profiles as ***R_eff_*** appears to adequately capture recent pandemic and seasonal influenza experience but may not apply to all emerging strains. At slightly higher ***R_0_*** = 1.80, our model findings also diverge dramatically indicating epidemic risk regardless of virus/immunity scenario assessed. We therefore emphasize findings at ***R_0_*** = 1.40 and present variation around that estimate but also highlight the need to better define influenza transmissibility characteristics generally and more specifically for emerging strains of interest.

As with all simulations, our findings are limited by model assumptions. Our platform is predicated on immunity profiles and a previously established network structure for the GVA–an urban region with characteristics that may not be generalizable to other settings or periods. We have assumed typical influenza characteristics and explored across a range of parameter assumptions further reconciled with surveillance observations. Because H3N2v is the descendant of an earlier ancestral human strain [32]–[33], the assumption of typical human influenza characteristics is reasonable, but unique zoonotic characteristics, additional variability or outlier possibilities cannot be ruled out. To account for stochastic effects we have averaged across multiple simulations but a degree of residual error must be acknowledged. Sero-protection estimates are subject to the usual caveats detailed above and, in addition, are based on non-random cross-sectional sero-sampling by ten-year age band rather than model-specified age categories. For ease and comparability, the same modeling approach was applied to each influenza strain, such that nuanced age-related differences between strains were not accommodated. Estimates of H3N2v sero-protection reflect accumulated cross-reactive antibody formed through prime-boost exposure to related strains and as such mostly represent prior, rather than current, social interactions and exposure opportunities. Within relevant age categories specified, it is assumed that accumulated influenza exposures would be similar between individuals. We thus varied immunity based on sero-protection estimates across age strata, but within each age stratum, we randomly assigned that immunity. Estimates of sero-protection we measured in the GVA in 2010 may differ by time and place. For the post-H1N1pdm09 profile, waning antibody, particularly vaccine-induced, as well as antigenic change in circulating virus must be taken into account for subsequent seasons. For H3N2v, the latter also applies and in addition, a cohort effect of accumulating susceptibility may become more influential as more children born after the mid-1990s, and lacking priming exposure to related strains, age into adolescence and adulthood. As that pool of susceptibility increases, the tipping point for epidemic spread may alter. In that regard, ongoing surveillance, sero-survey and simulation monitoring are warranted.

In summary, our simulations based on age-specific immunity and contact network interactions suggest H3N2v does not currently pose a substantial pandemic threat. If epidemics do occur, overall community attack rates are unlikely to exceed typical seasonal influenza experience. A greater proportion of young children could be affected and a proportion of the affected, young or old, will experience severe outcomes. Risk levels may change with time as additional cohorts of younger children without exposure to antigenically-related strains age into adolescence and adulthood. Ongoing monitoring, development of vaccine candidates and discussions related to optimal targeting strategies thus remain prudent. Our model assumptions and predictions appear robust as tested against surveillance observations for the humanized 2009 pandemic H1N1 virus and for recent seasonal influenza H3N2 experience. However, interpretation ultimately depends upon the accuracy of crucial epidemiologic characteristics–notably the serologic correlate of protection and transmissibility– essential features requiring urgent validation for risk assessment related not only to H3N2v but also other influenza viruses and applications of public health interest. Our combined in silico model- and observed data-based approach to assessing and quantifying the likelihood and magnitude of epidemic spread for novel pathogens could assist public health authorities in their planning, preparedness and rapid response activities. We thus encourage other scientists working in the field of infectious disease dynamics to further consider the impact of contact networks and immunity on the spread of newly emerging viruses with a view to informing real time risk assessment.

## Supporting Information

Figure S1 **Effect of immunity profile on the average number of effective contacts per week by virus scenario and age category.** This figure illustrates the average degree, i.e. the number of potentially infection-causing interpersonal contacts of individuals in a particular age group taking into account assigned immunity profiles.
(PDF)

Figure S2 **Age-stratified contact mixing matrices based on varying virus and immunity scenarios.** Panels display the contact mixing matrices for six virus/age/immunity scenarios. A contact-mixing matrix contains the colour-coded average number of contributory links, i.e. links between susceptible individuals.
(PDF)

Table S1 **Blended composite of sero-protection based on gradient of immunity defined as ½SPR40, ¾SPR80 and SPR160 with and without also incorporating ¼SPR20.**
(PDF)
