# Supplementary material for: H3N2v and Other Influenza Epidemic Risk Based on Age-Specific Estimates of Sero-Protection and Contact Network Interactions
Source: PLoS One. 2013 Jan 11;8(1):e54015. doi: 10.1371/journal.pone.0054015 (PMC3543419; doi:10.1371/journal.pone.0054015)
Supplement: Figure S1 — Effect of immunity profile on the average number of effective contacts per week by virus scenario and age category. This figure illustrates the average degree, i.e. the number of potentially infection-causing interpersonal contacts of individuals in a particular age group taking into account assigned immunity profiles. (PDF) [file pone.0054015.s001.pdf]

## Figure S1. Effect of immunity profile on the average number of effective contacts per week by virus scenario and age category

This figure illustrates the average degree, i.e. the number of potentially infection-causing interpersonal contacts of individuals in a particular age group taking into account assigned immunity profiles. Only contributory links are counted resulting in a decrease in degree for populations that are no longer completely susceptible. The contact mixing matrices, presented in Figure S2, show an age-stratified version of the average degree; in these matrices, a breakdown of the degree into the individual age groups is illustrated.

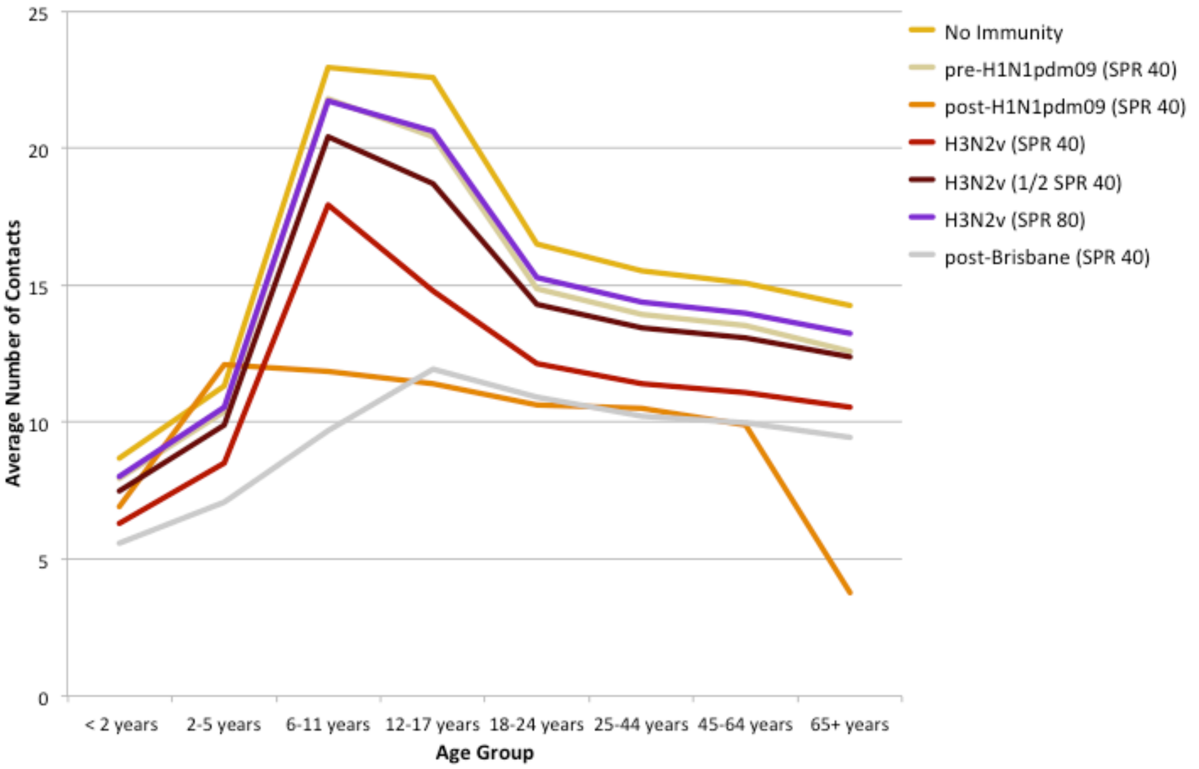


**SPR** = Sero-protection rate – defined as the proportion (%) considered sero-protected on the basis of having met or exceeded the specified antibody titre threshold
**pre-H1N1pdm09**: 2009 H1N1 pandemic virus; SPR presented based on PRE-pandemic antibody levels measured in 2009 or earlier
**post-H1N1pdm09**: 2009 H1N1 pandemic virus; SPR presented based on POST-pandemic antibody levels measured in fall 2010
**H3N2v**: swine-origin H3N2 variant strain; SPR presented based on antibody levels measured in sera collected in fall 2010
**post-Brisbane:** a contemporary seasonal human influenza H3N2 virus; SPR presented based on post-circulation antibody levels in sera collected in fall 2010
**SPR40**: the proportion considered sero-protected according to the standard hemagglutination inhibition (HI) titre threshold of 40
**½SPR40**: assumes half the individuals meeting SPR40 are considered sero-protected
**SPR80**: the proportion considered sero-protected according to a hemagglutination inhibition (HI) titre threshold of 80

Note that a blended composite of sero-protection based on a gradient of immunity defined as **¼**SPR20, ½SPR40, ¾SPR80 and 100% SPR160 was also explored but is not illustrated (see Table S1 and manuscript narrative).
