# Supplementary material for: H3N2v and Other Influenza Epidemic Risk Based on Age-Specific Estimates of Sero-Protection and Contact Network Interactions
Source: PLoS One. 2013 Jan 11;8(1):e54015. doi: 10.1371/journal.pone.0054015 (PMC3543419; doi:10.1371/journal.pone.0054015)

Figure S2. Age-stratified contact mixing matrices based on varying virus and immunity scenarios

The contact mixing matrices for six virus/age/immunity scenarios are illustrated. A contact-mixing matrix contains the average number of contributory links, i.e. links between susceptible individuals. In each row, the average number of links between individuals of one age group with another age group is colour-coded. Light colours refer to fewer links, whereas dark colours to a higher number of links. Different population sizes in an age category result in asymmetric contact mixing matrices.

Whereas H1N1pdm09 (Scenario pre-H1N1pdm09 SPR40) emerged into a mostly susceptible population in which the number of contributory links is similar to that of a population with no immunity, epidemic profiles are altered by population immunity as illustrated for the post-Brisbane SPR40 scenario. We explored H3N2v patterns across various age-specific immunity scenarios.

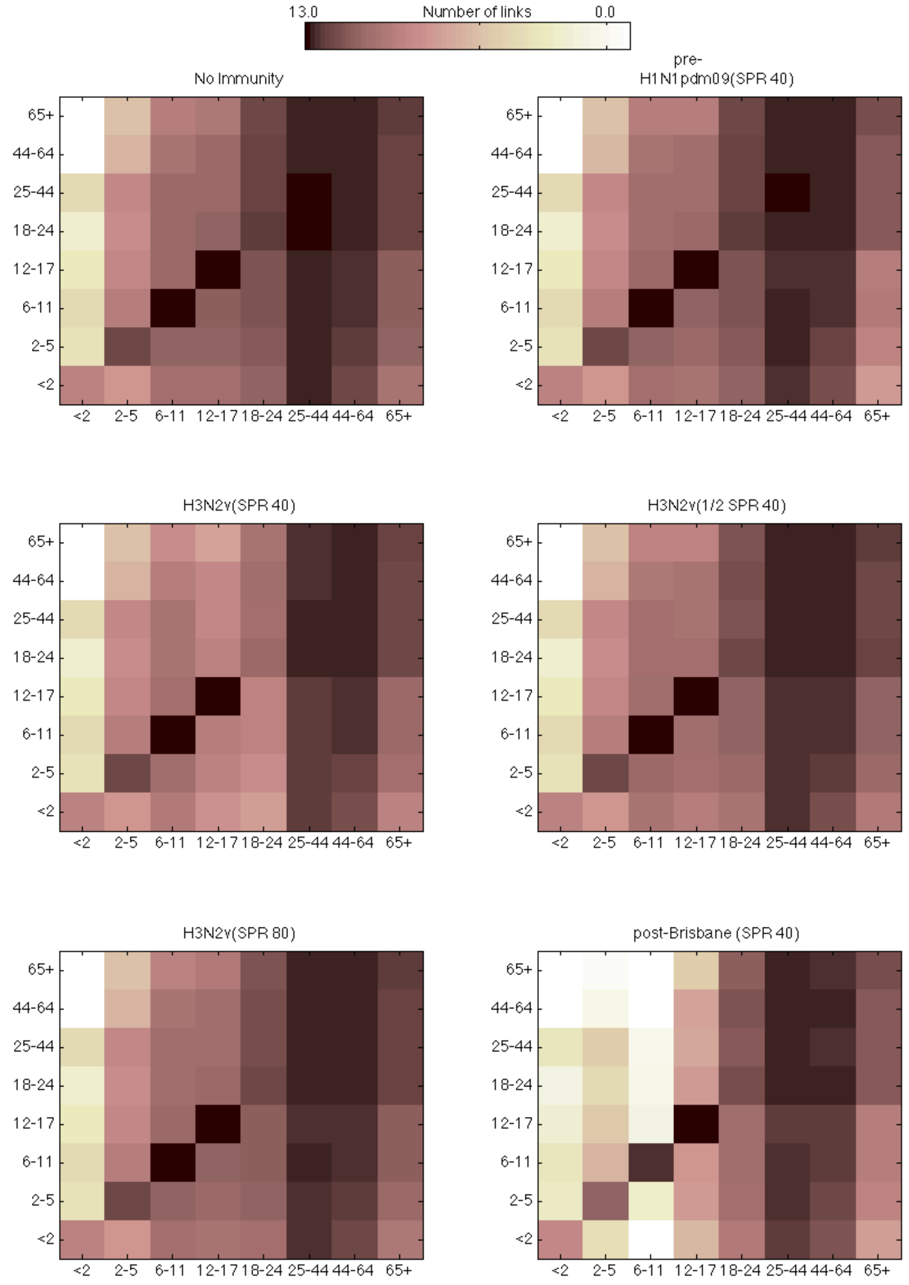

Supplement: Figure S2 — Age-stratified contact mixing matrices based on varying virus and immunity scenarios. Panels display the contact mixing matrices for six virus/age/immunity scenarios. A contact-mixing matrix contains the colour-coded average number of contributory links, i.e. links between susceptible individuals. (PDF) [file pone.0054015.s002.pdf]
