# Supplementary material for: H3N2v and Other Influenza Epidemic Risk Based on Age-Specific Estimates of Sero-Protection and Contact Network Interactions
Source: PLoS One. 2013 Jan 11;8(1):e54015. doi: 10.1371/journal.pone.0054015 (PMC3543419; doi:10.1371/journal.pone.0054015)
Supplement: Table S1 — Blended composite of sero-protection based on gradient of immunity defined as ½SPR40, ¾SPR80 and SPR160 with and without also incorporating ¼SPR20. (PDF) [file pone.0054015.s003.pdf]

Table S1. Blended composite of sero-protection based on gradient of immunity defined as ½SPR40, ¾SPR80 and SPR160 with and without also incorporating ¼SPR20

A. Not incorporating ¼SPR20

Age Categories (Years)	Proportion (%) considered sero-protected by virus scenario			
	Pre-H1N1pdm09	Post-H1N1pdm09	H3N2v	Post-Brisbane
**< 2**	0.0	39.1	0.0	5.0
**2-5**	0.6	57.5	0.0	32.1
**6-11**	0.5	43.3	9.8	57.2
**12-17**	5.8	39.2	23.6	35.1
**18-24**	1.6	18.3	38.5	23.4
**25-44**	5.0	18.0	20.9	17.2
**45-64**	2.7	8.6	3.2	14.8
**65+**	27.4	24.6	11.3	28.8

B. Incorporating ¼SPR20

Age Categories (Years)	Proportion (%) considered sero-protected by virus scenario			
	Pre-H1N1pdm09	Post-H1N1pdm09	H3N2v	Post-Brisbane
**< 2**	0.0	39.4	0.0	5.0
**2-5**	0.6	57.5	0.2	32.1
**6-11**	0.8	43.5	10.6	57.8
**12-17**	6.2	39.2	23.6	36.5
**18-24**	1.6	18.6	41.7	23.7
**25-44**	5.0	18.7	21.4	17.5
**45-64**	2.9	9.1	4.1	15.7
**65+**	28.3	25.1	12.0	29.4

SPR = Sero-protection rate – defined as **½**SPR40 (i.e. 50% of those with titre 40-79 protected), ¾SPR80, (i.e. 75% of those with titre 80-159 protected) and SPR160 (i.e. all with titre 160 or higher protected) without (A) and with (B) also incorporating ¼SPR20 (i.e. 25% with titre 20-39 protected).
pre-H1N1pdm09: 2009 H1N1 pandemic virus; SPR presented based on PRE-pandemic antibody levels measured in 2009 or earlier
post-H1N1pdm09: 2009 H1N1 pandemic virus; SPR presented based on POST-pandemic antibody levels measured in fall 2010
H3N2v: swine-origin H3N2 variant strain; SPR presented based on antibody levels measured in sera collected in fall 2010
post-Brisbane: a contemporary seasonal human influenza H3N2 virus; SPR presented based on post-circulation antibody levels in sera collected in fall 2010

VERSION: December 11, 2012
